# Analysis of Degenerative and Isthmic Lumbar Spondylolisthesis from the Difference of Pelvic Parameters and the Degree of Degeneration through Imaging Data

**DOI:** 10.3390/jpm13091420

**Published:** 2023-09-21

**Authors:** Zhide Liu, Guoyu Dai, Yong Cao, Chunyue Duan

**Affiliations:** 1 Department of Spine Surgery and Orthopaedics, Xiangya Hospital, Central South University, Xiangya Road 87, Changsha 410008, China198112297@csu.edu.cn (G.D.);; 2 National Clinical Research Center for Geriatric Disorders, Xiangya Hospital, Central South University, Xiangya Road 87, Changsha 410008, China; 3 Key Laboratory of Organ Injury, Aging and Regenerative Medicine of Hunan Province, Changsha 410008, China

**Keywords:** degenerative lumbar spondylolisthesis (DLS), isthmic lumbar spondylolisthesis (ILS), disc degeneration (DD), facet joint osteoarthritis (FJOA), paravertebral muscle (PVM), lumbar lordosis (LL), pelvic incidence (PI), pelvic tilt (PT), sacral slope (SS), L4 inclination angle (L4IA)

## Abstract

Background: In previous studies, many imaging analyses have been conducted to explore the changes in the intervertebral disc degeneration (DD), facet joint osteoarthritis (FJOA), L4 inclination angle (L4IA), pelvis-related parameters, lumbar lordosis (LL), and paravertebral muscle (PVM) in the occurrence and development of degenerative spinal diseases via measuring the X-ray, CT, and MRI data of clinical patients. However, few studies have quantitatively investigated the pelvic parameters and the degree of spine degeneration in patients with degenerative lumbar spondylolisthesis (DLS) and isthmic lumbar spondylolisthesis (ILS). This study discusses the changes in the imaging parameters of DLS, ILS, and a control group; explores the correlation between different measurement parameters; and discusses their risk factors. Methods: We evaluated 164 patients with single L4-L5 grade 1 level degenerative lumbar spondylolisthesis (DLS group), 161 patients with single L4-L5 grade 1 level isthmic lumbar spondylolisthesis (ILS group), and 164 patients with non-specific back pain (control group). The grades of DD and FJOA as well as the percentage of the fat infiltration area (%FIA) of multifidus muscle (MM) at the L4-L5 level were measured via CT and MRI. Lumbar lordosis (LL), pelvic incidence (PI), pelvic tilt (PT), the L4 inclination angle (L4IA), and sacral slope (SS) were measured via X-ray film, and the differences among the DLS group, ILS group, and control group were analyzed. Furthermore, the risk factors related to the incidences of the DLS and ILS groups were discussed. Results: First, the pelvis-related parameters of DLS and ILS patients were 51.91 ± 12.23 and 53.28 ± 11.12, respectively, while those of the control group were 40.13 ± 8.72 (*p*_1_ < 0.001, *p*_2_ < 0.001). Lumbar lordosis (LL) in DLS patients (39.34 ± 8.57) was significantly lower than in the control group (44.40 ± 11.79, *p* < 0.001). On the contrary, lumbar lordosis (LL) in the ILS group (55.16 ± 12.31) was significantly higher than in the control group (44.40 ± 11.79, *p* < 0.001). Secondly, the three groups of patients were characterized by significant variations in the L4 inclination angle (L4IA), disc degeneration (DD), facet joint osteoarthritis (FJOA), pelvis-related parameters, and paravertebral muscle (PVM) (*p* < 0.05). Finally, logistic regression suggests that the L4IA, FJOA, and PT may be risk factors for the occurrence of DLS, and the occurrence of ILS is correlated with the L4IA, FJOA, DD, PT, and LL. Conclusions: Compared with the control group, there are changes in pelvic parameters, the L4IA, LL, DD, FJOA, and PVM in DLS and ILS patients, and the degree is different. The parameters within the same group are related to each other, and DLS and ILS have different risk factors. The mechanical stability of the spine is affected by the parameter and angle changes, which may be of great significance for explaining the cause of spondylolisthesis, evaluating the health of the lumbar spine, and guiding the lifestyles of patients.

## 1. Introduction

Lumbar spondylolisthesis refers to the relative slip of a lumbar vertebral body relative to its lower vertebral body. The direction can be forward, backward, or lateral, of which forward is the most common. According to the etiology of lumbar spondylolisthesis, it can be divided into degenerative, vertebral arch isthmic, vertebral arch hypoplasia, traumatic, pathological, and iatrogenic. Degenerative lumbar spondylolisthesis (DLS) and isthmic lumbar spondylolisthesis (ILS) are the most common types in clinical practice [1]. DLS is a common spinal degeneration disease, often causing low back pain (LBP) and impacting quality of life [2]. DLS is a kind of spinal instability where a vertebral body slips over the one below without disrupting the pars interarticularis, which is different from isthmic spondylolisthesis; about 73% of DLS patients are at the L4-L5 level [3]. Many factors have been believed to cause the occurrence and development of DLS, associated with aspects such as disc degeneration, facet joint capsular tissues, ligamentous hypertrophy or buckling, and ineffectual muscular stabilization [4]. However, the exact etiology of DLS is still controversial, and there is a lack of sufficient evidence to support it. ILS is associated with interjoint defects. Spondylolisthesis occurs in 40% to 66% of patients with bilateral spondylolysis and is more common in men. The incidence of low back pain in patients with isthmic spondylolisthesis is relatively low, and most patients remain asymptomatic. The most common lumbar segment is L5-S1, followed by L4-L5. The cause of isthmic lumbar spondylolisthesis is thought to be multifactorial, for example, maximal weight bearing of the lumbar spine, a relatively weak anatomy, congenital dysplasia, hyperextended sports, and genetic factors [5].

In the past, spinal surgeons for lumbar spondylolisthesis focused on relieving symptoms of lower back pain and nerve compression, such as a neural decompression and obtaining a bony fusion. As spinal surgery developed, the concept of maintaining the mechanical stability of the spine and slowing down its degeneration has been emphasized as important for managing spinal disease. At the lumbar spine level, intervertebral discs, facet joints, and paravertebral muscles play an essential role in spinal stability and movement function. There have been many studies about the association between spine disorders and disc, facet joint, and paravertebral muscle degeneration. Chen et al. found that there was significantly lower disc height in patients with DLS than patients without lumbar spondylolisthesis [6]. Wang et al. found that the angle of facet joints could be a predictor of DLS [7]. Labelle H et al. found that adult patients with ILS generally have higher pelvic incidence, sacral slope, pelvic tilt, and lumbar lordosis [8]. These studies support that lumbar spondylolisthesis may be correlated with DD, FJOA, pelvis-related parameters, and PVM change. Nevertheless, few reports have performed a quantitative evaluation of changes in these factors in lumbar spondylolisthesis and the differences between DLS and ILS. The object of this study was to make a quantitative evaluation to describe the characteristics of DD, FJOA, pelvis-related parameters, and PVM change in patients with DLS and ILS, as well as to probe the mechanism of the onset and progression of lumbar spondylolisthesis in patients.

## 2. Materials and Methods

### 2.1. Subjects

This was a cross-sectional study in which 489 patients were enrolled. The DLS group included 164 patients with single L4-L5 grade 1 level DLS who were diagnosed at the Department of Spine Surgery at Xiangya Hospital of Central South University from February 2017 to December 2022. The ILS group included 161 patients with single L4-L5 grade 1 level isthmic lumbar spondylolisthesis, while the control group included 164 patients with non-specific back pain diagnosed at Xiangya Hospital from February 2017 to December 2022. The characteristics of the patients are summarized in Table 1.

### 2.2. Inclusion and Exclusion Criteria

All patients’ symptoms did not respond to conservative treatment for at least 6 months. Patients were excluded from the study if they met one or more of the following criteria, according to clinical and/or radiological data: (1) Multisegment DLS, ILS, and lumbar spinal stenosis. (2) Lumbar spondylolisthesis above grade 2. (3) Congenital deformity of the spine or adult degenerative scoliosis. (4) Spinal fracture, spinal tumor, and spinal tuberculosis. (5) Soft-tissue disease such as muscle injury and infection. (6) Received surgical treatment or brace treatment. (7) Lack of radiologic data.

### 2.3. Imaging Procedures

All of the patients underwent radiography, computerized tomography (CT), and magnetic resonance imaging (MRI). The MRI device used in this study was a 1.5-T MRI scanner (Siemens, Berlin, Germany), and the position of the participants was supine. The parameters of the scans were as follows: sagittal T1-weighted and T2-weighted images as well as axial T2-weighted images were obtained from T12 to the sacrum (matrix size: 224 × 384, slice thickness: 4 mm, and the repetition time (TR)/echo time (TE) for the T1-weighted images was 482 ms/10 ms, while the TR/TE for the T2-weighted images was 2450 ms/100 ms). The CT used in this study was a 256-slice scanner (Philips Brilliance; Philips, Eindhoven, The Netherlands) with a slice thickness of 0.625 mm. The radiography system used in this study was a 500 mA Siemens DR system (Siemens, Berlin, Germany) with an automatic exposure control system. All of the data were evaluated and collected by two independent doctors who have more than 10 years of work experience.

### 2.4. Image Analysis

#### 2.4.1. Lumbar Lordosis Angle, L4 Inclination Angle, and Pelvis-Related Parameters

**Figure 1:**
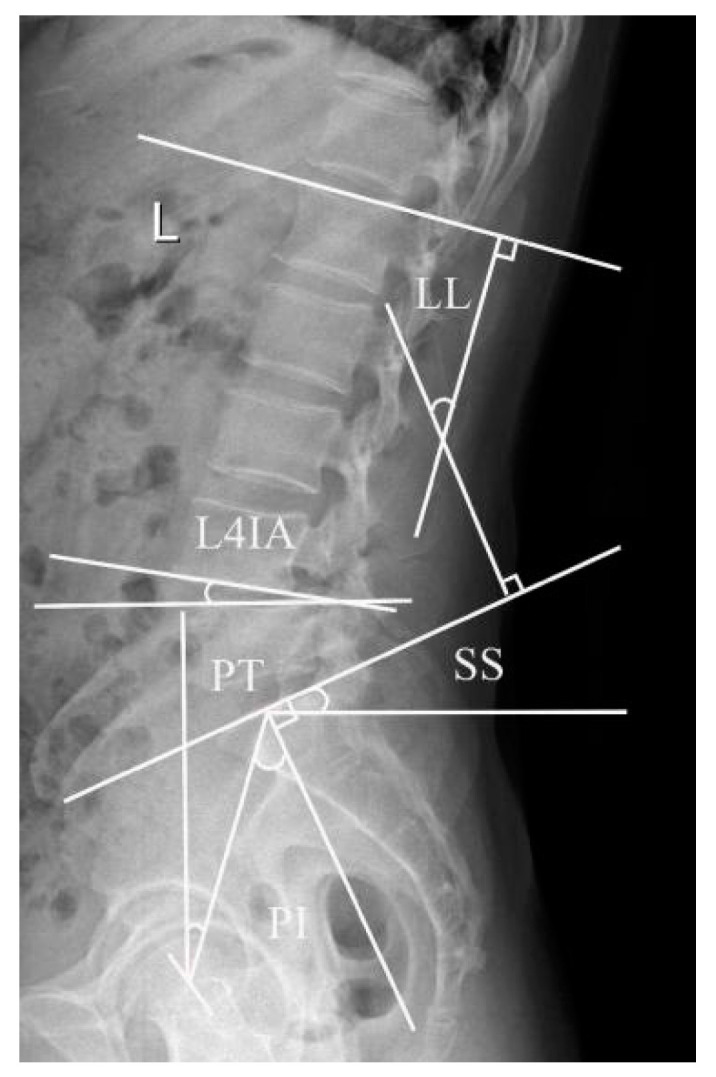
This illustration displays the pelvic incidence (PI), the sacral slope (SS), the pelvic tilt (PT), the lumbar lordosis (LL), and the L4 inclination angle (L4IA).

Lumbar lordosis (LL): The sagittal angle between the L1 upper endplate and the tangent of the S1 upper endplate. L4 inclination angle (L4IA): The intersection between the tangent line and the horizontal line at the lower margin of the fourth lumbar vertebra. Pelvic incidence (PI): The angle between the perpendicular to the upper sacral endplate at its midpoint and the line connecting this point to the femoral head axis. This is a morphological parameter that is considered to be a constant and has nothing to do with the spatial orientation of the pelvis. Sacral slope (SS): The angle between the horizontal and the upper sacral endplate. It is a positional parameter, which varies according to the pelvis position. Pelvic tilt (PT): The angle between the vertical and the line passing through the midpoint of the sacral plate to the axis of the femoral head. It is also a position parameter (Figure 1). 

#### 2.4.2. Disc Degeneration Grading 

**Figure 2:**
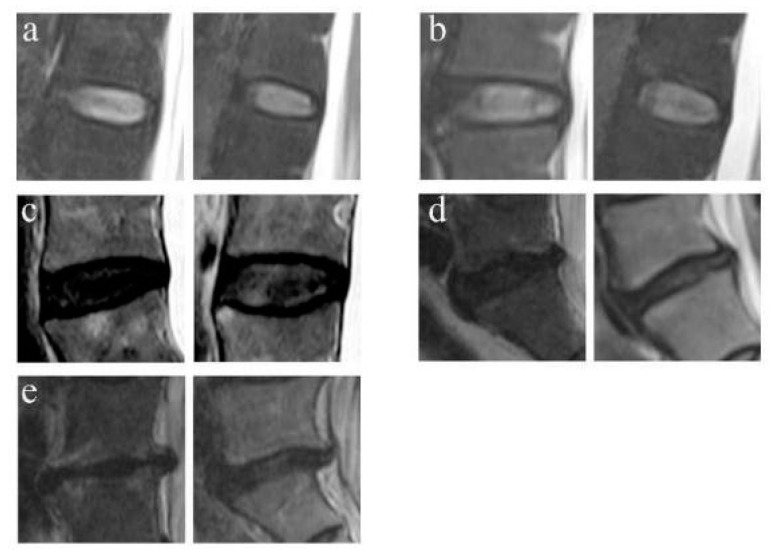
Example of Pfirrmann grades for the LDD. (**a**) Grade Ⅰ: normal discs. (**b**) Grade Ⅱ: the mild degeneration discs with structure inhomogeneous with/without horizontal bands. (**c**) Grade Ⅲ: the moderate degeneration discs with gray structure, can not distinguish the nucleus and annulus, intermediate signal intensity, and normal/slightly decreased disc height. (**d**) Grade Ⅳ: the severe degeneration disc with gray/black structure, the distinction of nucleus and annulus is lost, intermediate/hypointense signal intensity, and normal/moderately decreased disc height. (**e**) Grade Ⅴ: End-stage degeneration disc with black structure, the distinction of nucleus and annulus is lost, hypointense signal intensity, and collapsed disc space.

The LDD was classified into 5 grades by using the criterion of Pfirrmann et al. on T2-weighted MRI [9]. Grade 1 corresponds to a normal disc and grade 5 corresponds to a terminal degenerative disc (Figure 2). 

#### 2.4.3. FJOA Grading 

**Figure 3:**
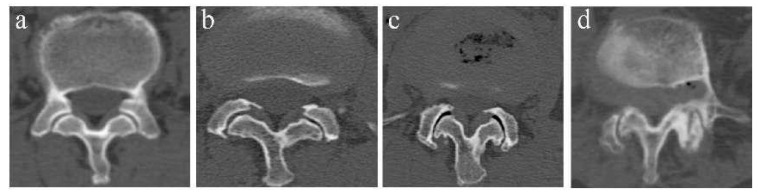
Example of FJOA on CT. (**a**) Grade 0: normal facet joint. (**b**) Grade 1: mild osteoarthritis facet joint with narrow space and small osteophytes. (**c**) Grade 2: moderate osteoarthritis with sclerosis or subchondral erosions. (**d**) Grade 3: severe osteoarthritis with marked osteophyte.

The FJOA was classified into 4 grades via the criterion of Pathria et al. [10] on CT. Grade 0 corresponds to a normal facet joint and grade 3 corresponds to a severely degenerated joint (Figure 3).

#### 2.4.4. Evaluation of PVM Change 

**Figure 4:**
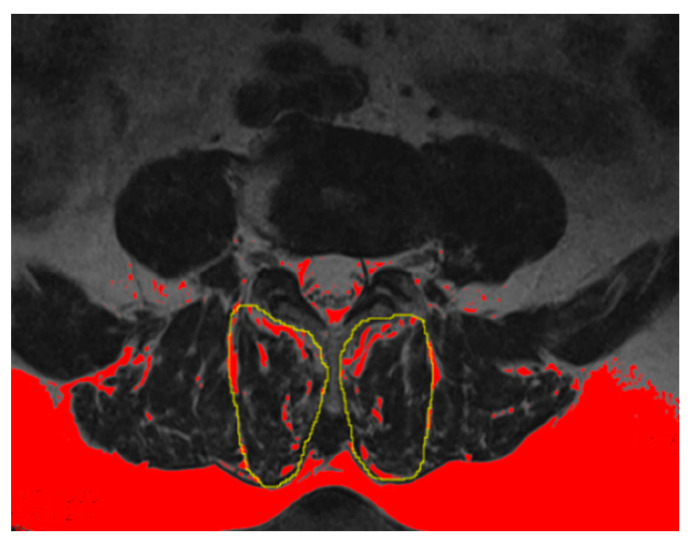
Example of fat tissue (red color) of the MM showed in the ImageJ software (Version 2.0.0, National Institutes of Health, Bethesda, MD, USA).

The multifidus muscle (MM) has been the most studied and commonly used measurement with which to evaluate the fat infiltration and atrophy of PVM [11,12]. The percentage of the fat infiltration area (%FIA) was measured via axial T2-weighed imaging at the L4-L5 disc level by using ImageJ software (Version 2.0.0, National Institutes of Health, Bethesda, MD, USA). First, we converted each image into a grayscale 8-bit image. We then outlined the region of the multifidus muscle using the threshold technique and utilized a “default” and “dark background” method to obtain the value of the threshold automatically. Last, we calculated the %FIA; the red area in the 8-bit image was the fat tissue, and the fat tissue divided by the region of the multifidus muscle was the %FIA (Figure 4) [13].

### 2.5. Statistical Analysis 

The analyses were performed using SPSS software version 26.0 (IBM SPSS, Armonk, NY, USA), and *p*-values of <0.05 were considered statistically significant. The normal distribution of analysis results was assessed using the Shapiro–Wilk test. The difference in the sex ratio between the three groups was assessed by using the Chi-square test. The BMI, age, and %FIA of the MM of the three groups were assessed by using a nonparametric test. The FJOA and DD scores among the three groups were assessed by using the Wilcoxon rank sum test. Logistic regression analysis was used to analyze the possible risk factors of DLS and ILS. The reliability of analysis results measured within and between observers was assessed using the intra-group correlation coefficient (ICC). 

## 3. Results

A total of 489 patients were recruited and evaluated in this retrospective study. There were 59 males and 105 females in the control group, 49 males and 115 females in the DLS group, and 53 males and 108 females in the ILS group. There were no significant differences in gender, BMI, and age among the three groups (Table 1).

### 3.1. Lumbar Lordosis Angle, L4 Inclination Angle, and Pelvis-Related Parameters

Regarding pelvis-related parameters, the average pelvic incidence of DLS patients and ILS patients was 51.91 ± 12.23 and 53.28 ± 11.12, respectively, while that of the control group was 40.13 ± 8.72 (significant difference). The lumbar lordosis (LL) of the DLS patients (39.34 ± 8.57) was significantly lower than that of the control group (44.40 ± 11.79, *p* < 0.05). On the contrary, lumbar lordosis in the ILS group was larger than that in the control group (55.16 ± 12.31 and 44.40 ± 11.79, respectively, *p* = 0.000). Compared with the control group (26.33 ± 6.96), SS in the DLS group decreased (22.28 ± 7.25, *p* < 0.05), while SS in the ILS group increased (30.20 ± 7.95, *p* < 0.05). However, for the control PT (13.81 ± 8.87), both the DLS group (29.64 ± 11.37, *p* = 0.000) and the ILS group (23.07 ± 8.60, *p* = 0.000) showed an increasing trend. The L4IAs of the DLS (14.87 ± 4.02, *p* = 0.000) and ILS (11.89 ± 8.59, *p* = 0.000) groups were significantly different to that of the control group (6.27 ± 2.15), and there were also differences between the two groups (*p* < 0.001) (Table 2).

### 3.2. Disc Degeneration Grading

The degree of intervertebral disc degeneration among the control group, DLS group, and ILS group was statistically different (*p*_1_ < 0.001, *p*_2_ < 0.001, and *p*_3_ < 0.001) (Table 3).

#### 3.2.1. FJOA Grading

There were statistical differences in the grade of FJOA among the control group, DLS group, and ILS group (*p*_1_ < 0.001, *p*_2_ < 0.001, and *p*_3_ < 0.001) (Table 4).

#### 3.2.2. Evaluation of PVM Change

The mean %FIA of the MM in the control group was 19.71 ± 9.05, which was significantly different from that in the DLS group (21.89 ± 7.51, *p* < 0.05) and ILS group (25.29 ± 8.12, *p* < 0.001) (Table 5).

#### 3.2.3. Logistic Regression Analysis of DLS and ILS Predictors

Logistic regression showed that the L4IA (β: 0.850, *p* < 0.001), FJOA (β: 1.420, *p* = 0.002), and PT (β: 0.153, *p* = 0.002) may be the risk factors for the occurrence of DLS (Table 6). The occurrence of ILS is related to the L4IA (β: 0.172, *p* < 0.001), FJOA (β: 0.686, *p* = 0.006), DD (β: 1.672, *p* < 0.001), PT (β: 0.110, *p* < 0.001), and LL (β: 0.057, *p* < 0.001) (Table 7).

## 4. Discussion

### 4.1. Degenerative Lumbar Spondylolisthesis and Isthmic Spondylolisthesis

DLS and ILS are different in terms of etiology, development mechanisms, natural development, and treatment. ILS is defined as the loss of fibers in the isthmus of the posterior pedicle, which leads to the protrusion of the upper vertebral body and the separation of the anterior surface of the vertebral body from the neural arch. The main mechanism is considered to be repetitive and periodic back and forth flexion, especially the stress fractures caused by extension [14]. It will lead to a series of degenerative changes in the spine, which will lead to nerve compression, serious pain, and neurological symptoms, so surgery is needed. Degeneration is caused by degenerative changes and the instability of the lumbar spine, which lead to hypertrophy of the bones and soft tissues, thus causing back pain and neurological symptoms [15]. If this condition does not respond to conservative treatment and the pain persists, surgery is required [2]. There is little research on the degenerative characteristics of these two types of spondylolisthesis, especially for polyfidus fat infiltration and slippage risk factors. In this study, we evaluated the degree of degeneration and related factors by analyzing plain film, CT, and MRI.

### 4.2. Spinopelvic Parameters

A large number of measurement parameters and evaluation criteria are used for the sagittal balance measurement of the spine. Currently, the most widely accepted pelvic parameter system is the PI system, including LL [16]. PI is a fixed constant after bone development and maturity, which can reflect the shape of the pelvis. PI represents the algebraic sum of SS and PT: PI = SS + PT. However, some studies show that PI is not constant throughout life, and it may increase to a certain extent after individual long-stage fusion or after the age of 75 [17,18]. PT describes the relationship between the pelvis and the femoral head. The increase in PT means that the pelvis rotates backward with the femoral head as the midpoint and the opening of the pelvis becomes upward-facing. The opposite change occurs when PT decreases [19]. The size of SS/PT is generally used to reflect pelvic posture [20]. LL is also an important force to maintain the sagittal balance of the spine. It does not change with age. It is mainly influenced by the shape of the vertebral body and discs. It may also affect the facet joints and the forward tilt of the pelvis [21].

In the current study, the PI of the DLS and ILS groups was significantly higher than that of the control group, indicating that a pelvic morphology with high PI may be a predisposing factor for lumbar spondylolisthesis. The comparison of PT and SS among the three groups showed that, compared with the control group, patients with lumbar spondylolisthesis had pelvic retroversion, especially in the DLS group. When a slip occurs, it is necessary to re-establish the spinal balance by adjusting the force line, so reducing LL and pelvic backward tilt becomes the best compensation mechanism. Isthmic spondylolisthesis patients with isthmic spondylolisthesis have higher LL, which may be due to the high shear stress in the joint area, which may lead to a joint defect (spondylolysis) and eventually develop into ILS. Meanwhile, higher LL releases the pressure on the pelvis from sagittal instability of the spine, so the corresponding pelvic backward tilt is alleviated. Moreover, this study suggests that the L4IA may be related to the occurrence of a slip. Previous studies have suggested that the stress on the fourth lumbar vertebra was mainly divided into shear stress parallel to the endplate and compressive stress perpendicular to the endplate [22]. The increase in the tilt angle of the fourth lumbar vertebra will increase its shear stress, thus increasing the forward driving force of the vertebral body, increasing the chance of spondylolisthesis.

### 4.3. Disc Degeneration

The Pfirrmann classification was used for intervertebral disc degeneration, which was more obvious in the DLS and ILS groups than in the control group. Intervertebral disc degeneration is a risk factor for lumbar spondylolisthesis; it is considered an “endogenous stabilizing system” for the stability of the human spine. In ILS, interjoint defects do not allow the posterior spinal elements to fully share the load, so the intervertebral disc space is subjected to disproportionately higher stresses [23]. Due to the deficiency of the posterior element, the weight of the body will be applied vertically to the spine, and at the same time, a shear moment will be applied to the intervertebral disc at the slip-off position. The increase in vertical gravity increases the shear force and subsequently the disc degenerates at a higher rate, resulting in a degeneration characterized by a loss of disc height and annulus relaxation [24]. Eventually, intervertebral disc degeneration and ILS promote each other, resulting in a vicious cycle.

### 4.4. Facet Joint Osteoarthritis

The lateral shear stress caused by lumbar spondylolisthesis can lead to increased stress on the facet joints. According to the research, facet joints carry about 3–25% of the axial compressive load [25]. Generally, failure of biomechanical function would lead to FJOA [26]. There may be two reasonable explanations for the occurrence of FJOA in DLS: First, via an increase in the degree of DS, the effective stressed area and height of the disc decreased, which would increase the axial compressive load of the facet joint and the chance of impingement on the lamina or the pars interarticularis. Second, facet joint subluxation. With the progression of DLS, we found that the inferior articular process of the L4 will displace forward at the L4-L5 facet joint, followed by the effective stressed area of the facet joint decreasing. Compared with the intervertebral disc, the facet joints have a smaller contact area and are more sensitive to the progression of DLS, while for the ILS group, the mechanical stress on the facet joint may be decreased because of the interarticular defect, so the progress of facet joint degeneration is relatively less than that of DSL.

### 4.5. Percentage of the Fat Infiltration Area (%FIA) of the Multifidus Muscle (MM)

Previous cross-sectional studies on adults have found that the severity of fat infiltration in paraspinal muscles is closely related to low back pain, spinal dysfunction, and intervertebral disc degeneration [27]. This relationship is not affected by body mass index, type of work, or level of leisure time physical activity [28]. The lumbar multifidus muscle is an important structure in the maintenance of spinal flexion balance. During spinal movement, it provides greater rigidity than other paraspinal muscles. A decline in multifidus muscle function will lead to decreased lumbar stability [29,30]. In this study, polyfidus fat infiltration is statistically different in the DLS and ILS groups compared with the control group, which indicated that muscle fat infiltration may be a risk factor for lumbar spondylolisthesis. For ILS, intervertebral disc degeneration is significant, and applying more gravity and motion to the back of the spine may lead to more muscle fat infiltration. In addition, research shows that the MM is only innervated by a single spinal root at the same level [11]. With the development of DLS, intervertebral disc degeneration and facet joints degeneration or hypertrophy will lead to nerve root compression, and denervation will accelerate the %FIA of the MM. 

### 4.6. Correlation between Different Imaging Parameters in the Two Groups

For DLS, as we increase in age, the intervertebral disc experiences degenerative changes. The loss of fluid and elasticity in the intervertebral disc and the decrease in its height lead to vertebral instability and finally lead to the translation and sliding of the vertebral body. Such a loss of intervertebral disc height and the changes in mechanical properties will lead to posterior facet joint degeneration and low back pain by increasing the load on the facet joints [31]. Therefore, the degeneration of the intervertebral disc is the initial factor of facet joint degeneration. The instability of the intervertebral disc and facet joints increases the load of the posterior muscles and increases intramuscular fat infiltration. The function of polyfidus muscle tissue decreased and the function of maintaining sagittal plane balance weakened. However, the results show that patients with high PI and high LL have less intervertebral disc degeneration, which may possibly be because they contribute to the stability of the sagittal plane, reduce the transverse shear forces of the intervertebral disc and facet joints, and slow down the degeneration.

For ILS, both gravity and posture forces acting on the upright spine exert pressure on the interarticular region, making it vulnerable to injury. This is more obvious for patients with high LL. At the same time, the muscles behind the vertebral body exert too much force to minimize the shear moment, leading to compensatory lordosis over spondylolismus. In addition, in order to maintain the sagittal balance of the body, the patient compensates by pelvic rotation [32]. Therefore, the SS/PT of ILS patients reduced and the pelvis tilted backward, but the larger LL of ILS patients weakens the compensatory effect of pelvic rotation.

### 4.7. Possible Risk Factors for DLS and ILS

Logistic regression analysis suggested that the L4IA, FJOA, and PT may be risk factors for the occurrence of DLS; however, previous studies have shown that high PI, DD, and polyfidus fatty infiltration are risk factors for DLS, possibly because high PI suggests pelvic compensatory ability, as indicated by the risk factor PT. It may also be the case that the sample is small and no meaningful statistical results are seen. It is possible that DD and muscle fat infiltration are not direct risk factors of DLS, but indirect factors after degeneration of the L4IA and facet joint degeneration; therefore, PI, DD, and polyfidus fat infiltration are also important influencing factors of DLS. Considering that the cause of the disease is not entirely due to degeneration, the L4IA and LL can be considered direct risk factors, while FJOA and DD are more regarded as indirect risk factors, forming a vicious circle with the progress of ILS.

### 4.8. Limitations of This Study

A limitation of this study is that the study of the characteristics of the disease is limited to a certain hospital, and the patients are not random. This study is only a retrospective study of patients with back pain, and asymptomatic patients should also be included. In addition, only patients with one episode of a slip were included, and the findings are not universal. This study involves relatively few patients and only considered radiological parameters. In addition, it is necessary to further follow up whether a patient has undergone surgery and the changes in postoperative imaging parameters to determine the correlation between imaging parameters and clinical results.

## 5. Conclusions

Compared with the control group, the changes in LL, DD, FJOA, and PVM were observed in DLS and ILS groups, which indicated that lumbar spondylolisthesis may be a manifestation of late spinal degeneration and may also be related to different congenital spinal and pelvic morphologies. Meanwhile, lumbar spondylolisthesis further accelerates the degeneration of the spinal structure, and the degeneration of different parts affects and promotes other parts. The human body adjusts itself to these changes. It is of great significance to guide patient rehabilitation and patient lifestyles.

## Figures and Tables

**Table 1:** Demographic characteristics of both groups.

Variable	Control Group	DLS Group	ILS Group	Statistics	*p*-Value
Cases	164	164	161		
Male/female	59/105	49/115	53/108	χ^2^ = 1.381	0.501
Age (years)	61.0 ± 8.7	62.3 ± 6.3	60.5 ± 6.9	t = 2.588	0.076
BMI (kg/m^2^)	24.75 ± 2.38	24.02 ± 2.72	24.51 ± 4.13	t = 2.012	0.1348

**Table 2:** Comparison of spinopelvic parameters in DLS, ILS and control groups.

Spinopelvic Parameters	Control Group	DLS Group	ILS Group	*p*_1_	*p*_2_	*p*_3_
PI (°)	40.13 ± 8.72	51.91 ± 12.23	53.28 ± 11.12	<0.001 *	<0.001 *	0.29
SS (°)	26.33 ± 6.96	22.28 ± 7.25	30.20 ± 7.95	<0.001 *	<0.001 *	<0.001 *
PT (°)	13.81 ± 8.87	29.64 ± 11.37	23.07 ± 8.60	<0.001 *	<0.001 *	<0.001 *
LL (°)	44.40 ± 11.79	39.34 ± 8.57	55.16 ± 12.31	<0.001 *	<0.001 *	<0.001 *
L4IA (°)	6.27 ± 2.15	14.87 ± 4.02	11.89 ± 8.59	<0.001 *	<0.001 *	<0.001 *

*p*_1_: *p* value between Control group and DLS group; *p*_2_: *p* value between Control group and ILS group; *p*_3_: *p* value between DLS group and ILS group; * means there is a statistical difference.

**Table 3:** The grades of Disc degeneration in DLS, ILS and control groups at the L4-5 level.

DD	Control Group	DLS Group	ILS Group	Statistics	*p*-Value
Grade 1	8	0	0	Z_1_ = 5.90 Z_2_ = 11.18 Z_3_ = 7.79	*p*_1_ < 0.001 * p_2_ < 0.001 * p_3_ < 0.001 *
Grade 2	51	11	3		
Grade 3	65	82	24		
Grade 4	36	59	87		
Grade 5	4	12	47		

*p*_1_: *p* value between Control group and DLS group; *p*_2_: *p* value between Control group and ILS group; *p*_3_: *p* value between DLS group and ILS group; Z_1_: Z value between Control group and DLS group; Z_2_: Z value between Control group and ILS group; Z_3_: Z value between DLS group and ILS group; * means there is a statistical difference.

**Table 4:** The grades of FJOA in DLS, ILS and control groups at the L4-5 level.

FJOA	Control Group	DLS Group	ILS Group	Statistics	*p*-Value
Grade 0	64	10	54	Z_1_ = 15.86 Z_2_ = 10.17 Z_3_ = 4.06	*p*_1_ < 0.001 * p_2_ < 0.001 * p_3_ < 0.001 *
Grade 1	237	89	85		
Grade 2	25	154	126		
Grade 3	2	75	57		

*p*_1_: *p* value between Control group and DLS group; *p*_2_: *p* value between Control group and ILS group; *p*_3_: *p* value between DLS group and ILS group; Z_1_: Z value between Control group and DLS group; Z_2_: Z value between Control group and ILS group; Z_3_: Z value between DLS group and ILS group; * means there is a statistical difference.

**Table 5:** Evaluation of %FIA of MM in DLS, ILS and control groups.

L4-5	%FIA			Statistics	*p*-Value
	Control Group	DLS Group	ILS Group		
Mean	19.71 ± 9.05	21.89 ± 7.51	25.29 ± 8.12	t_1_ = 2.37 t_2_ = 5.85 t_3_ = 3.92	*p*_1_ < 0.05 * p_2_ < 0.001 * p_3_ < 0.001 *

t_1_: t value between Control group and DLS group; t_2_: t value between Control group and ILS group; t_3_: t value between DLS group and ILS group; * means there is a statistical difference.

**Table 6:** Logistic regression analysis of the predictors for DLS.

Risk	β	95%CI	OR	*p*-Value
L4IA	0.850	1.832–2.988	2.340	0.000 *
FJOA	1.420	1.713–9.986	4.136	0.002 *
PT	0.153	1.058–1.283	1.166	0.002 *

CI: Confidence Interval; OR: Odds Ratio; * means there is a statistical difference.

**Table 7:** Logistic regression analysis of the predictors for ILS.

Risk	β	95%CI	OR	*p*-Value
L4IA	0.172	1.113–1.267	1.188	0.000 *
FJOA	0.686	1.218–3.237	1.986	0.006 *
DD	1.672	3.083–9.182	5.321	0.000 *
PT	0.110	1.050–1.188	1.117	0.000 *
LL	0.057	1.023–1.096	1.059	0.001 *

CI: Confidence Interval; OR: Odds Ratio; * means there is a statistical difference.

## Data Availability

The data supporting our findings can be found in the article.
